# 

**Published:** 1935

**Authors:** 


### The Medical Library of the University of Bristol,

WITH WHICH IS INCORPORATED

**The Library of the Bristol Medico-Chirurgical Society.**

---

*The following donations have been received since the publication of the list in March,* 1935.

June, 1935.

| | |
|---|---|
| Dr. Buntaro Adachi (1) .. .. .. .. | 1 volume. |
| Bengal Medical College (2) .. .. .. | 1 ,, |
| Brompton Hospital for Consumption and Diseases of the Chest (3) .. .. .. .. | 1 ,, |
| Thomas Carwardine, Esq. (4) .. .. .. | 10 volumes. |

| | |
|---|---|
| University of Durham College of Medicine (5) .. | 1 volume. |
| General Medical Council (6) .. .. .. | 3 volumes. |
| Henry Phipps Institute (University of Pennsylvania) (7) .. .. .. .. | 1 volume. |
| Professor E. W. Hey Groves (8) .. .. | 10 volumes. |
| Dr. F. S. Hunter (9) .. .. .. .. | 1 volume. |
| Michell Clarke Fund (10) .. .. .. | 1 ,, |
| Professor C. Lloyd Morgan (11) .. .. | 1 ,, |
| Otago University Medical School (12) .. .. | 2 volumes. |
| Dr. George Parker (13) .. .. .. .. | 1 volume. |
| Professor C. Bruce Perry (14) .. .. .. | 5 volumes. |
| Professor R. S. S. Statham (15) .. .. | 6 ,, |
| Dr. P. Watson-Williams (16) .. .. .. | 5 ,, |
| Messrs. John Wright & Sons (17) .. .. | 1 volume. |

Unbound periodicals have also been received from Professor E. W. Hey Groves, Professor C. Bruce Perry and Dr. J. Odery Symes.

### THE ONE HUNDRED AND FIFTY-FIRST LIST OF BOOKS.

The figures in round brackets refer to the figures after the names of the donors. The books to which no such figures are attached, have either been bought from the Library Fund or received through the *Journal.*

| | | |
|---|---|---|
| **Abernethy, J.** .. .. | *Surgical Observations on the Constitutional Origin and Treatment of Local Diseases ; and on Aneurisms* .. .. .. .. (15) | 1809 |
| **Abernethy, J.** .. .. | *Surgical Observations on Diseases Resembling Syphilis* .. .. .. .. .. .. (15) | 1810 |
| **Adachi, B.** .. .. | *Das Venensystem der Japaner* .. .. (1) | 1933 |
| **Bailey, H., and Love, R. J. M.** | *A Short Practice of Surgery* 2nd Ed. | 1935 |
| **Basset, A.** .. .. .. | *Le Genou* .. .. .. .. .. .. .. .. .. (8) | 1932 |
| **Basset, A., et Miliaret, J.** | *L'épaule* .. .. .. .. .. .. .. .. (8) | 1934 |
| **Bendien, S. G. T.** .. | *Specific Changes in Blood Serum.* Translated by Piney .. .. .. .. .. .. .. .. .. | 1931 |
| **Bland-Sutton, J.** .. | *The Bradshaw Lecture on Misplaced and Missing Organs* .. .. .. .. .. .. (4) | 1917 |
| **Bland-Sutton, J.** .. | *Cancer Clinically Considered* .. .. .. (4) | 1909 |
| **Bland-Sutton, J.** .. | *Essays on Hysterectomy* .. .. .. .. (4) | 1905 |
| **Bland-Sutton, J.** .. | *Selected Lectures and Essays* .. .. .. (4) | [1920] |
| **Blechmann, G.** .. .. | *La Ponction Epigastrique de Macfan* .. (14) | 1913 |
| **Böhler, L.** .. .. .. | *The Treatment of Fractures.* 4th Ed. Translated from the German by E. W. Hey Groves .. .. .. .. .. .. .. .. | 1935 |

**Bostock, J.** .. .. .. *An Elementary System of Physiology.* 3 Vols. [Vol. 1=2nd Ed.] .. .. .. .. (15) 1826–1828

**Breul, K., ed.** .. .. *A German and English Dictionary* .. .. [1934]

**Brockbank, E. M.** .. *A Centenary History of the Manchester Medical Society* .. .. .. .. .. .. (13) 1934

**Bull, H. C. H.** .. .. *X-Ray Interpretation* .. .. .. .. .. .. 1935

**Byrne, J. G.** .. .. *Clinical Studies on the Physiology of the Eye* .. .. .. .. .. .. .. .. .. .. 1934

*Centenary of the Bengal Medical College* .. .. .. .. .. .. .. (2) [1935]

**Cope, Z.** .. .. .. .. *The Early Diagnosis of the Acute Abdomen* 6th Ed. 1932

**Daniels, D. W.** .. *An Outline of Surgery for Nurses* .. .. 1933

**Davidson, M.** .. .. *A Practical Manual of the Diseases of the Chest* .. .. .. .. .. .. .. .. .. .. 1935

**Davis, G. G.** .. .. *Applied Anatomy* .. .. .. .. 9th Ed. (8) [1934]

**Douglas, J.** .. .. *Myographiae Comparatae Specimen* .. (4) 1707

**Elmer, W. P., and Rose, W. D.** *Physical Diagnosis* .. 7th Ed. 1935

**Fagge, C. H.** .. .. *The Acute Abdomen* .. .. .. .. .. .. 1932

**Fisher, A. G. T.** .. *Internal Derangements of the Knee-joint* 2nd Ed. (8) —

**Gaitskell, C. E.** .. *Radiological Terminology* .. .. .. .. (8) 1935

**Graham, E. A., ed.** *Practical Medicine Series,* 1931 : *General Surgery* .. .. .. .. .. .. .. .. .. [1932]

**Gravier, L.** .. .. *L'Alternance du Cœur* .. .. .. .. .. (14) 1914

**Hammond, T. E.** .. *Principles in the Treatment of Inflammation* 1934

**Hartmann, A.** .. .. *Diseases of the Ear.* Translated by Erskine (16) 1887

**Hohmann, G.** .. .. *Fuss und Bein, ihre Erkrankungen und deren Behandlung* .. .. .. 2te Auflage (8) 1934

**Hurst, A. F.** .. .. *The Sensibility of the Alimentary Canal* (4) 1911

**Hutchison, R., and Hunter, D.** *Clinical Methods* .. 10th Ed. 1935

**Inman, F. W.** .. .. *Biological Politics* .. .. .. .. .. .. .. .. 1935

**Jeaffreson, J. C.** .. *A Book About Doctors.* Vol. I. .. .. (4) 1860

**Kanavel, A. B.** .. *Infections of the Hand* .. .. 6th Ed. (8) 1934

**Kefalas, A.** .. .. *What of the Child?* .. .. .. .. .. .. .. .. 1934

**Keith, A.** .. .. .. *Illustrated Guide to the Museum of the R.C.S. of England* .. .. .. .. .. .. .. .. (4) 1910

**Ladd, G. T., and Woodworth, R. S.** *Physiological Psychology* .. (11) 1911

**Langley, J. N.** .. .. *The Autonomic Nervous System.* Vol. I. (4) 1921

**Layton, T. B.** .. .. *Catalogue of the Onodi Collection in the Museum of the R.C.S.* .. .. .. .. .. .. (17) 1934

**Lermoyez, M.** .. .. *Thérapeutique des maladies des fosses nasales.* 2 Vols. .. .. .. .. .. .. .. (16) 1896

**Lloyd-Williams, K. G.** *Anæsthesia and Analgesia in Labour* .. .. 1934

**McAusland, S.** .. .. *The Cure of Hæmorrhoids and Varicose Veins* (9) 1934

**McCann, F. J.** .. .. *The Treatment of Common Female Ailments* 3rd Ed. 1934

| | | |
|---|---|---|
| **McGregor, A. L.** .. | *Synopsis of Surgical Anatomy* 2nd Ed. (8) | 1934 |
| **Miller, A. A.** .. .. | *Bendien's Diagnostic Method for Cancer* .. | 1931 |
| **Moussons, Barbier, etc.** | *Maladies du Cœur et des Vaisseaux du Nez, du Larynx, des Bronches, des Poumons, des Plèvres et du Médiastin.* No. 4 of "*La Pratique des maladies des enfants*" (14) | 1911 |
| *Nouveau traité de médécine.* Fasc XX. .. .. .. .. .. .. (10) | | 1935 |
| **Osler, W.** .. .. .. | *The Principles and Practice of Medicine* (16) | 1892 |
| **Penrose, L. S.** .. .. | *The Influence of Heredity on Disease* .. .. | 1934 |
| **Platt, R.** .. .. .. | *Nephritis and Allied Diseases* .. .. .. | 1934 |
| **Price-Jones, C.** .. .. | *Red Blood-cell Diameters* .. .. .. .. .. | 1933 |
| **Price-Jones, C.** .. .. | *Blood Pictures* .. .. .. .. .. .. 3rd Ed. | 1933 |
| **Pye, W.** .. .. .. | *Elementary Bandaging and Surgical Dressing.* Revised by A. J. Cokkinis. 15th Ed. (8) | 1933 |
| **Ransome, A.** .. .. | *The Treatment of Phthisis* .. .. .. (16) | 1896 |
| **Rugg-Gunn, A.** .. | *Diseases of the Eye* .. .. .. .. .. .. .. .. | 1933 |
| **Souttar, H. S.** .. | *Radium and Cancer* .. .. .. .. .. .. | 1934 |
| **Spek, J. van der** .. | *On Osteogenic Sarcoma* .. .. .. .. .. (8) | [1933] |
| **Sully, J.** .. .. .. | *Outlines of Psychology.* .. New Ed. (4) | 1894 |
| **Tawara, S.** .. .. | *Das Reizleitungssystem des Säugetierherzens* (14) | 1906 |
| **Thomas, E. W. C.** .. | *A Synopsis of Forensic Medicine and Toxicology* .. .. .. .. .. .. .. .. .. | 1933 |
| **Tucker, W. E.** .. | *Injuries and Their Treatment* .. .. .. | 1935 |
| **Turner, G. G., and Arnison, W. D.** | *The Newcastle-upon-Tyne School of Medicine,* 1834–1934 .. .. .. (5) | 1934 |
| **Vaquez, H., and Bordet, E.** | *Le cœur et l'aorte* .. .. 3rd Ed. (14) | 1920 |
| **Velpeau, A. L. M.** .. | *Embryologie ou ovologie humaine* .. .. (15) | 1834 |
| **Watson, F.**.. .. .. | *Hugh Owen Thomas*.. .. .. .. .. .. .. .. | 1934 |
| **Whitnall, S. E.** .. | *Anatomy of the Human Orbit* .. 2nd Ed. | 1933 |

### TRANSACTIONS, REPORTS, JOURNALS, ETC.

| | | |
|---|---|---|
| Brompton Hospital Reports. Vol. III. .. .. .. .. .. .. | (3) | 1934 |
| Dentists' Register .. .. .. .. .. .. .. .. .. .. .. .. .. .. .. .. | (6) | 1935 |
| Medical and Dental Students' Register, 1934 .. .. .. .. .. | (6) | [1935] |
| Medical Annual .. .. .. .. .. .. .. .. .. .. .. .. .. .. .. .. .. | | 1935 |
| Medical Register .. .. .. .. .. .. .. .. .. .. .. .. .. .. .. .. | (6) | 1935 |
| Proceedings of the University of Otago Medical School, No. 12, and Supplement. Ed. by D. W. Carmalt-Jones .. .. .. .. .. | (12) | 1935 |
| Quarterly Cumulative Index Medicus. Vol. XVI. .. .. .. .. .. | | [1935] |
| Surgical Clinics of North America. Vol. 15, Nos. 1 and 2 .. .. | | 1935 |
| University of Pennsylvania : Twenty-fifth Report of the Henry Phipps Institute for the Study, Treatment and Prevention of Tuberculosis, 1934 .. .. .. .. .. .. .. .. .. .. .. .. .. .. .. .. .. .. | | 1934 |

### Publications Received

From Messrs. Arnold & Co.:

*Midwifery.* By Ten Teachers. 5th Edition. Price 18s.

From Messrs. John Bale, Sons and Danielsson Ltd.:

*Ophthalmology in General Practice.* By O. Gayer Morgan. Price 2s. 6d.

From Messrs. A. and C. Black Ltd.:

*Forensic Medicine.* By D. J. A. Kerr, M.D. Price 15s.

From Messrs. Cassell and Co. Ltd.:

*A Handbook of Midwifery.* By Sir C. Berkeley, M.A., M.C., M.D. 9th Edition. Price 8s.

From Messrs. H. K. Lewis & Co. Ltd.:

*Collected Papers of St. Mark's Hospital, London, including a History of the Hospital.* Centenary volume, compiled by the Medical Committee. Price 30s.

*Simple Instructions for Diabetic Patients.* By Dorothy C. Hare, M.D. 2nd Edition. Price 1s.

*Standard Classified Nomenclature of Disease.* Edited by H. B. Logie. 2nd Edition. Price 15s.

*A Text-Book on the Nursing and Diseases of Sick Children, for Nurses.* Edited by A. Moncrieff, M.D., F.R.C.P. 2nd Edition. Price 15s.

*Gould's Medical Dictionary.* Edited by R. J. E. Scott, M.A., M.D., and C. V. Brownlow. 4th Edition. Price 30s.

*Theory and Practice of Nursing.* By M. A. Gullan. 4th Edition. 1935. 9s.

From Medical Superintendent (Norwood Sanatorium), Rendlesham Hall, Woodbridge.

*Report of the Norwood Sanatorium for the Years* 1932, 1933 and 1934.

From Oxford University Press:

*Hugh Owen Thomas: his Principles and Practice.* By D. McCrae Aitken, M.A., M.B., Ch.B., F.R.C.S. 1935. Price 12s. 6d.

From The Student Christian Movement Press:

*Religion and Psychotherapy.* By A. G. Ikin, M.A., M.Sc. Price 3s. 6d.

From Messrs. John Wright & Sons Ltd.:

*The Treatment of Fractures.* By L. Böhler, M.D. English translation by E. W. Hey Groves. 4th Edition. Price 42s.

*British Journal of Surgery.* Vol. 22, No. 88. 1935. Annual Subscription, 42s.

*Medical Annual,* 1935. 53rd year. Price 20s.

### Local Medical Notes

---

Opening of the New Nurses' Home at the Bristol Royal Hospital for Women and Children.

The new Nurses' Home of the Children's Hospital was opened on the 27th of March, 1935, by Lady Apsley. In the absence of the President through illness the Chair was taken by Mrs. Pretorius, Chairman of the Ladies' Auxiliary, who was supported by the Committee and a large number of distinguished guests.

The old Nurses' Home was an ancient building when first occupied by the Hospital in 1857, and had become entirely unsuitable for its purpose. The new Home has been built in the grounds of the Hospital, and is more than 200 feet above sea-level. The ground floor is devoted to sitting-rooms and studies for the sisters and nurses, which open on to the garden. The bedrooms occupy three floors and command extensive views over the city and surrounding country. The furnishing has been provided by the Ladies' Auxiliary, whose Chairman, Mrs. Pretorius, made the whole of the eighty-seven rugs and mats used in the Home to her own beautiful designs.

The Home has been built to the designs of Messrs. Oatley and Lawrence at a cost of £20,000, and forms the second part of a rebuilding scheme. The third part of this will be the erection of a new Out-patient Department, which has yet to be carried out when circumstances permit. When this has been completed the Hospital will have been brought up to the requirements of modern medical science.

---

**The Long Fox Memorial Lecture.**—The twenty-fourth Long Fox Memorial Lecture will be delivered by Dr. A. MacDonald Critchley, M.B., F.R.C.P., on Tuesday, October 29th, at 5 p.m., in the University. The subject will be announced later.

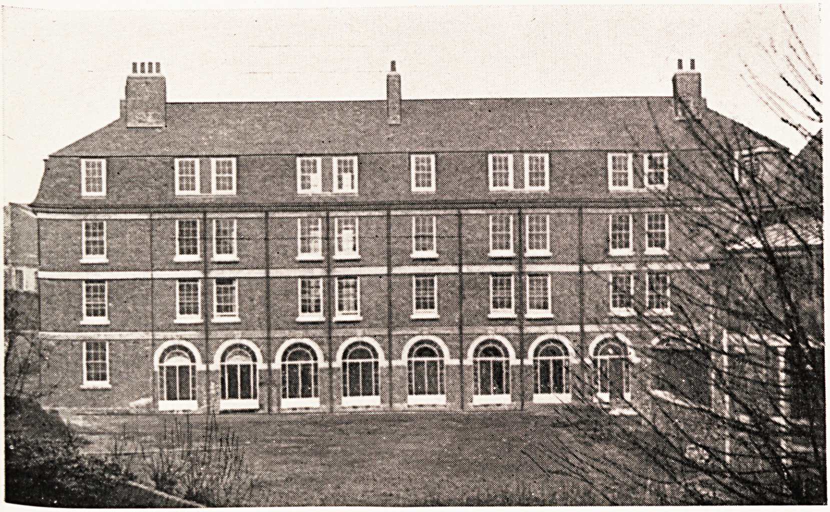

THE NEW NURSES' HOME AT THE BRISTOL ROYAL HOSPITAL FOR WOMEN AND CHILDREN.

APPOINTMENTS.

**Bristol Royal Infirmary.**—L. A. Moore, M.B., Ch.B., M.R.C.S., L.R.C.P., Honorary Anæsthetist. A. L. Eyre-Brooke, M.R.C.S., L.R.C.P., Senior Resident Officer.

**Bristol General Hospital.**—G. F. Langley, M.B., Ch.B., M.R.C.S., L.R.C.P., Senior Resident Medical Officer.

**Bristol Royal Hospital for Sick Children and Women.**—Elizabeth M. Cooke, M.D., M.R.C.P. Lond., Assistant Honorary Physician.

EXAMINATION RESULTS.

**University of Bristol.**—Students of the University have recently passed the following examinations :—

M.D.—*Pass with Distinction :* J. F. Coates. *Pass :* H. F. M. Finzel, J. P. P. Stock.

M.B., Ch.B..—*Second Examination.* (*Section II.*) *Pass :* K. J. V. Carlson, A. A. K. Datta, Emily G. Hamlyn, M. M. Lewis, P. C. C. Phelps, J. S. Richardson

M.B., Ch.B.—*Final Examination.* (*Section I.*) *Pass :* R. D. Caesar, Coralie W. Rendle-Short, P. Zimmering, Centa Alkan, Ursula G. Hewitt.

M.B., Ch.B.—*Final Examination.* (*Section II.*) *Pass* (*With Second Class Honours*) *:* Kathleen G. Brimelow, S. D. Loxton (with Distinction in Surgery). *Pass :* F. C. Collingwood, A. E. Jowett, B. Ridgway, A. W. Woolley.

B.D.S.—*Second Examination. Pass :* D. R. Stevens.

B.D.S. — *Final Examination. Pass* (*With Second Class Honours*) *:* J. W. E. Snawdon.

L.D.S.*—*Pass :* L. H. Barker-Tufft, H. C. Davey, K. A. Gordon-Ralph, G. S. Hewitt, E. L. Manton, L. N. Weale.

L.D.S.—*First Professional Examination. Pass :* F. S. S. Baguley, D. C. Bodenham, C. F. Howell, C. C. Moore, A. J. H. Orchard, D. M. Sanders, P. H. S. Paine.

L.D.S.—*Second Professional Examination. Pass :* M. J. Banbury, R. A. Hilton, R. J. Lee, L. K. A. S. Oxley, M. G. Davies, J. C. F. Rolls, H. W. Williams, J. G. Windmill.

L.D.S.—*Final Professional Examination. Pass :* R. E. Buchan, J. G. Clancy, B. E. Ffrench, Frances M. Orton, D. E. Royal, L. E. Scull.

* Preliminary Science Examination.

**University of Cambridge.**—*Third Examination for Medical and Surgical Degrees, Easter Term,* 1935. *Part II. Pass :* C. J. F. Coombs.

**University of London.**—M.B., B.S. (*Part I.*).—*Pass :* R. G. Boyd, Marjorie O. Dunster, Betty Fox.

**Conjoint Board.** — M.R.C.S., L.R.C.P. *Pass : In Physiology :* A. L. T. Beddoe. *In Pharmacology and Materia Medica :* F. D. Mowat, Mabel L. Glenny, J. H. Bulleid. *In Medicine :* A. W. Woolley, A. E. Jowett,* E. J. Perks. *In Medicine and Surgery :* F. C. Collingwood, Kathleen G. Brimelow.* *In Midwifery :* W. H. Hayes, Ursula W. Wood. *In Surgery and Midwifery :* N. P. Eskell. *In Surgery :* Irene G. Hamilton, T. H. Taylor.

**Society of Apothecaries of London.** — L.S.A. — *Final Examination.—Pass : In Surgery and Forensic Medicine :* K. P. Pauli. *In Surgery :* L. E. Sealy. *In Surgery, Medicine and Forensic Medicine :* S. Roberts.

* Qualified.

